# Heterogeneity in signaled active avoidance learning: substantive and methodological relevance of diversity in instrumental defensive responses to threat cues

**DOI:** 10.3389/fnsys.2014.00179

**Published:** 2014-09-24

**Authors:** Isaac R. Galatzer-Levy, Justin Moscarello, Esther M. Blessing, JoAnna Klein, Christopher K. Cain, Joseph E. LeDoux

**Affiliations:** ^1^Department of Psychiatry, New York University School of MedicineNew York, NY, USA; ^2^Department of Arts and Sciences, Center for Neural Science, New York UniversityNew York, NY, USA; ^3^Nathan Klein InstituteOrangeburg, SC, USA

**Keywords:** signaled active avoidance, fear conditioning, threat conditioning, Posttraumatic Stress Disorder (PTSD), heterogeneity, latent growth mixture modeling, resilience

## Abstract

Individuals exposed to traumatic stressors follow divergent patterns including resilience and chronic stress. However, researchers utilizing animal models that examine learned or instrumental threat responses thought to have translational relevance for Posttraumatic Stress Disorder (PTSD) and resilience typically use central tendency statistics that assume population homogeneity. This approach potentially overlooks fundamental differences that can explain human diversity in response to traumatic stressors. The current study tests this assumption by identifying and replicating common heterogeneous patterns of response to signaled active avoidance (AA) training. In this paradigm, rats are trained to prevent an aversive outcome (shock) by performing a learned instrumental behavior (shuttling between chambers) during the presentation of a conditioned threat cue (tone). We test the hypothesis that heterogeneous trajectories of threat avoidance provide more accurate model fit compared to a single mean trajectory in two separate studies. Study 1 conducted 3 days of signaled AA training (*n* = 81 animals) and study 2 conducted 5 days of training (*n* = 186 animals). We found that four trajectories in both samples provided the strongest model fit. Identified populations included animals that acquired and retained avoidance behavior on the first day (Rapid Avoiders: 22 and 25%); those who never successfully acquired avoidance (Non-Avoiders; 20 and 16%); a modal class who acquired avoidance over 3 days (Modal Avoiders; 37 and 50%); and a population who demonstrated a slow pattern of avoidance, failed to fully acquire avoidance in study 1 and did acquire avoidance on days 4 and 5 in study 2 (Slow Avoiders; 22.0 and 9%). With the exception of the Slow Avoiders in Study 1, populations that acquired demonstrated rapid step-like increases leading to asymptotic levels of avoidance. These findings indicate that avoidance responses are heterogeneous in a way that may be informative for understanding both resilience and PTSD as well as the nature of instrumental behavior acquisition. Characterizing heterogeneous populations based on their response to threat cues would increase the accuracy and translatability of such models and potentially lead to new discoveries that explain diversity in instrumental defensive responses.

## Introduction

Animal models of stress are thought to provide information about the course and etiology of stress psychopathology such as Posttraumatic Stress Disorder (PTSD) (Yehuda and Antelman, 1993). Animal studies typically examine the mean response to threat challenge paradigms. However, a key feature of stress and trauma responses in humans is marked heterogeneity where only a minority of individuals develops significant and prolonged symptomatology (Bonanno, 2004; Yehuda and Ledoux, 2007; Galatzer-Levy et al., 2013a). Recently, researchers have begun to disaggregate heterogeneous populations of animals based on their behavioral response to acquired aversive cues. To date studies have identified distinct populations based on their rate and ability to extinguish learned threat (fear) cues (Bush et al., 2007; Cowansage et al., 2013; Galatzer-Levy et al., 2013b) as well as their ability to initiate instrumental behaviors to terminate such cues (Choi et al., 2010). Importantly distinct neurobiological mechanisms have been identified that differentiate subpopulations both in studies of threat extinction (Cowansage et al., 2013) and instrumental responses (Choi et al., 2010). Such an approach has significant promise for the identification social and neurobiological characteristics that influence the development of qualitatively distinct behavioral phenotypes.

Studies of Pavlovian conditioning have been formative in elucidating the neural mechanisms underlying conditioned defensive reactions (e.g., Johansen et al., 2011; Ledoux, 2014). Abnormal functioning of these neural mechanisms may underlie stress psychopathology (Yehuda and Ledoux, 2007). The initiation of situation-specific instrumental behaviors can ameliorate conditioned defensive reaction to threat cues such as freezing (Cain and LeDoux, 2008; Choi et al., 2010; Moscarello and Ledoux, 2013), just as the initiation of situation-specific active coping behaviors ameliorate the potential negative psychological effects of dangerous or harmful traumatic events (Gross and Thompson, 2007; Hartley and Phelps, 2010; Bonanno and Burton, 2013). Such behavioral outputs are thought to result from a complex interplay between afferent and efferent neurocircuitry governing arousal, threat learning, motivation, and habit formation. Characterizing individual differences in instrumental behaviors in response to threat and harm among animals exposed to identical experimental conditions can provide information about normal and abnormal functioning of this circuitry. Ultimately, this can facilitate neurobiology research of abnormal stress responses such as PTSD and healthy responses such as resilience.

Signaled active avoidance (AA), which combines sequential Pavlovian and instrumental conditioning (Mowrer and Lamoreaux, 1946), involves an active behavioral response to conditioned threat. In a typical experiment, rats are trained to shuttle across a divided chamber during auditory CS presentation, causing termination of the CS and omission of the footshock US (Choi et al., 2010; Ledoux, 2014). Importantly, while AA requires Pavlovian learning to encode threat, the transition to successful instrumental avoidance requires active suppression of freezing (Lázaro-Muñoz et al., 2010; Moscarello and Ledoux, 2013), an innate defensive response to a Pavlovian CS (Blanchard and Blanchard, 1969; Fanselow and Poulos, 2005).

Animals do not uniformly learn signaled AA. A subset of animals will not acquire the avoidance response, referred to as “Poor Avoiders” (Choi et al., 2010). Several studies have exploited this heterogeneity to investigate neural mechanisms that mediate competition between Pavlovian and instrumental memories during AA training (Choi et al., 2010; Lázaro-Muñoz et al., 2010; Martinez et al., 2013). Poor Avoiders also show increased Pavlovian freezing, and AA performance is restored in these animals by lesions of the central amygdala (CeA), a region that is essential for conditioned threat reactions (Choi et al., 2010; Lázaro-Muñoz et al., 2010). In a recent study, Martinez et al. (2013) compared brain c-Fos expression following AA training between good vs. poor AA avoiders, and found differences in amygdala-PFC circuits (Martinez et al., 2013) similar to those identified in good vs. poor extinguishers (Hefner et al., 2008).

These findings demonstrate that distinct subpopulations can be disaggregated to identify neurobiological mechanisms mediating distinctive profiles of avoidance-related behavior (Martinez et al., 2013). This approach may be particularly relevant to PTSD and other anxiety disorders, which occur in a minority of individuals (Kessler et al., 2005), and involve persistent and maladaptive threat responses, leading to increased vigilance and intrusive fear memories (Mahan and Ressler, 2012). Animal models of threat extinction have revealed neural mechanisms that translate to clinical findings, and are now central to current concepts of PTSD diagnosis and treatment (Parsons and Ressler, 2013). Active avoidance holds significant promise for understanding the functional interactions between circuits governing defense, arousal, reinforcement, motivation and control, which together instantiate behavior.

While distinct sub-populations can be disaggregated using cut-off scores based on behavioral responses, this does not provide evidence that such populations are truly present in the data. Only one study to date has attempted to empirically determine if such threat challenges produce distinct behavioral phenotypes (Galatzer-Levy et al., 2013b). This work utilized Latent Growth Mixture Modeling (LGMM) to statistically test for population heterogeneity in threat extinction learning over successive trials. LGMM provides a method to empirically identify heterogeneous latent classes distinguished by their pattern of change over time. Results of this study indicated that multiple homogeneous subpopulations in an overall heterogeneous population better fit the data than a single population. Identified populations included those who rapidly extinguished, those who slowly extinguished, and those who failed to extinguish Pavlovian reactions to the CS (Galatzer-Levy et al., 2013b), a pattern consistent with the heterogeneity in response trajectories following human trauma exposure, both in shape and proportion (Galatzer-Levy et al., 2013a). In clinical studies, LGMM techniques have been used to identify heterogeneous trajectories of symptom and stress response among individuals exposed to significant life stressors and traumatic events (Galatzer-Levy et al., 2011, 2012, 2013c, 2014; Bonanno et al., 2012a,b; Galatzer-Levy and Bonanno, 2012). Importantly, LGMM does not require *a priori* hypotheses of bimodal good vs. poor avoiders, but provides a statistical method for empirically determining the number and shape of trajectories that best fit the data, and a framework for testing hypotheses related to that heterogeneity (Del Boca, 2004). Thus LGM provides the opportunity to empirically identify and characterize those trajectories that best fit the data.

While discernable populations may be identified using cluster analytic techniques such as LGMM, it is important to determine if these populations are distinct behavioral phenotypes, or simply statistical anomalies. If the trajectories are valid, other behaviors typically associated with good or poor performance should also be differentiated by AA population membership. Previous evidence also indicates that animals that perform poorly during signaled AA training also demonstrate decreased inter- trial exploratory behavior freezing (Vicens-Costa et al., 2011). Thus, animals that demonstrate greater active avoidance will likely demonstrate greater inter- trial exploratory behavior.

In the current investigation, we test the hypothesis that heterogeneous patterns of signaled AA, as measured in an auditory two-way shuttling paradigm, can be identified and replicated using an LGMM approach. We also apply LGMM to simultaneously collected data on inter-trial crossing responses (ITRs), i.e., the number of times animals cross between divided chambers in between AA trials, which is a measure of inter-trial exploratory behavior. We predict that rapid avoiders identified by LGM should also show increased ITRs.

## Methods

### Animals

Subjects were 267 naïve male Sprague-Dawley rats (Hilltop Laboratories) weighing 250–300 g at the time of arrival. Animals were used in two separate AA studies. Study 1 (*n* = 81 rats) consisted of 3 days of signaled AA training while Study 2 (*n* = 186 rats) utilized identical procedures with training extended through 5 days. Rats were individually housed in plastic tubs with *ad libitum* access to food and water, and kept on a 12 h light/dark cycle (lights on at 8 AM). All procedures were approved by the NYU University Animal Welfare Committee. Animals in Study 1 had intracranial guide cannula implants as previously described (Moscarello and Ledoux, 2013) while animals in Study 2 did not.

### Apparatus

#### Signaled active avoidance apparatus

Signaled active avoidance training occurred in 6 identical Plexiglas and metal rectangular shuttle boxes (50.8 × 25.4 × 30.5 cm, LWH) separated into two equal compartments by a metal divider placed halfway along the length of the chamber (Coulbourn Instruments). A passage in the divider (8 × 9 cm, WH) allowed animals to move freely between compartments. The floor was comprised of conductive stainless steel bars. The CS was a 5 kHz, 70 db tone delivered via two speakers mounted on opposite walls of the chamber. The US was a 0.7 mA footshock administered via the floor by a scrambled shocker. The chamber was lit by two 0.5 W light bulbs, one in each compartment. The shuttle box was housed within a larger sound-attenuating cubicle.

Shuttling (movement from one compartment to the other) was monitored by two infrared arrays, each comprised of 5 emitter-detector pairs, located on either side of the metal divider. Sessions were also recorded on DVD by a pair of black and white infrared cameras, one in each compartment.

#### Signaled active avoidance training

On the day prior to the initiation of training, all animals were habituated to the shuttle box for 1 h. Shuttling between compartments was recorded as a measure of baseline activity. Twenty-four hours later the first of 3 or 5 consecutive daily avoidance-training sessions began. Each session started with a 5 min acclimation period in which no stimuli were presented. The 1st trial of the 1st session was a Pavlovian trial—a 15 s tone CS preceded a 1 s foot shock US regardless of whether the animal performed the avoidance response (shuttling) during CS presentation. This allowed all animals to acquire the Pavlovian contingency at the same point in training. All subsequent trials were avoidance trials. The CS lasted a maximum of 15 s and was followed immediately by a US lasting a maximum of 15 s. The shock begins immediately after the 15 s CS (if the rat fails to shuttle during the CS) and remains on until an escape shuttle occurs or 15 additional seconds elapse. If the animal shuttled during CS presentation, the tone terminated immediately, and the US was not delivered; this was scored as an avoidance response. Each session was comprised of 30 avoidance CSs with a varying inter-trial interval that was, on average, 120 s. Both CS and US duration depended on the behavior of the animals. If the rat shuttled during the CS presentation, it immediately terminated and thus the CS was less than 15 s. If no shuttle occurred during the CS, then the shock was presented until an escape shuttle occurred or until an additional 15 s elapsed. Thus, if the rat escaped the US then the US presentation was less than 15 s. There were no minimum CS or US durations programmed, only maximums. Avoidance responses were recorded as number of successful shuttles in response to the CS. Inter-trial shuttling responses (ITRs) were recorded simply as the number of non-CS shuttles per session.

### Data analytic approach

The current study attempts to identify latent (not directly observable) classes of active avoidance acquisition using LGMM. LGMM allows for the empirical exploration of underlying heterogeneity that may otherwise be treated as error when assuming population homogeneity across the sample under study (Del Boca, 2004). An advantage of the LGMM framework is that it provides tests to statistically compare the number of classes and other parameters (Muthen, 2003). LGMM uses a nested model approach where progressively complex models are compared statistically. In this context, the null model is a single linear trajectory characterized by the population mean. Key criteria for model selection are reductions across nested comparisons in the Information Criteria (IC) [the Bayesian Information Criterion (BIC), sample-size adjusted Bayesian Information Criterion (SSBIC), Aikaike Information Criterion (AIC) (Schwartz, 1978; Bozdogan, 1987; Sclove, 1987)], fit statistics [the Lo-Mendell-Rubin likelihood test (LRT), Bootstrap Likelihood Ratio Test (BLRT)], as well as parsimony and interpretability (Nylund, 2007). The IC specifically provides an index for model selection from a finite set of nested models. During model identification one can increase the likelihood function simply by adding more parameters. However, this can result in overfitting. The various IC's are indices that balance model complexity with fit by adding a penalty for added parameters to prevent selection of overfit models. The different IC's are closely related mathematically with small distinctions that result in better performance under different circumstances. As such, it is recommended to attend to all IC indices (Nylund, 2007).

LGMM methods, as well as related methods that utilize fixed effects such as including Latent Class Growth Analysis (LCGA), are useful for identifying and studying homogeneous stress response patterns without reliance on a priori assumptions to define cutoff scores for populations (Galatzer-Levy and Bryant, 2013).

Specifically LGMM methods test whether the population under study is composed of a mixture of discrete distributions characterized as classes of individuals who share profile of growth across measurement point, with class membership determined by parameters including the intercept, slope and other model specific parameters (Curran and Hussong, 2003). Consistent with other methods that utilize maximum likelihood estimation to identify models (though other methods such as Markov Chain Monte Carlo estimation can also be utilized), models may be identified that are only “local solutions” meaning that the model is only accurate in part of the data or a subset of the data. To guard against this, large numbers of random starting values are utilized to identify a solution that replicates across subjects resulting in a “global solution” (Duncan et al., 2006; Kline, 2011). This is conceptually similar to cross-validation methods that identify a solution in one random portion of the data and validate it in a different random portion of the data though it is not explicitly a cross-validation technique.

LGMM has been applied to a wide variety of behaviors in which it is not parsimonious to assume one population defined by a single continuous distribution, including drinking behavior among college students (Greenbaum et al., 2005), childhood aggression (Schaeffer et al., 2003), developmental learning trajectories (Boscardin, 2008), as well as posttraumatic stress in response to military combat (Bonanno et al., 2012b) trauma exposure among police (Galatzer-Levy et al., 2011, 2013c, 2014), emergency medical interventions (Deroon-Cassini, 2010; Galatzer-Levy et al., 2013a) and recently patterns of fear extinction in rats (Galatzer-Levy et al., 2013b).

In the current study, a piecewise modeling approach was utilized so that unique avoidance slopes for each day by class could be identified. Each piece covers three time points, separately capturing each day of training, and with a single intercept representing the number of successful escapes on the first set of trials. Within a piecewise model, multiple progressive linear slopes are modeled in the place of a single slope across time points allowing for information about the time frame of change to be captured without adding significant model complexity (Flora, 2008). We sought a model that was parsimonious, interpretable and that demonstrated lower values on the information criterion indices, and a significant p value for the BLRT and LMRT. We also examined entropy to assess the likelihood that individual rats were conforming to the modeled trajectories. Entropy in this context is a measure of correct classification into modeled parameters. As identified classes are modeled parameters, not cases per say, it is possible that individuals do not conform well to the parameters that are identified. Entropy provides an estimate of how well the data conforms to the modeled parameters. Entropy ranges from 0 to 1 with 1 indicating perfect classification. When entropy is low, it is inappropriate to save probable class assignment for analysis outside of the LGMM because significant error is introduced do to misclassification. Study 1 utilized fixed effects for the intercept and slopes because the relatively small sample size limits the ability to examine free parameters. Study 2 utilized fixed effects only for slopes 2 through 5. Fixing effects aids in model convergence but precludes analysis of covariates that can explain the random variability in these parameters. In the current study, we were primarily interested in identifying classes rather than testing predictors associated with the parameters so fixing these effects did not limit the current study.

### Data analysis

First, the 30 consecutive daily trials were binned so that each consecutive score represents the mean number of avoidance responses across 10 trials. As such, Study 1 consists of 9 consecutive avoidance scores and Study 2 consists of 15 avoidance scores per rat. Data for ITRs came from study 2 and consisted of 5 measurement points capturing total number of ITRs per session. Using Mplus 6.12 (Muthen and Muthen, 2006), LGMM was employed to identify heterogeneous trajectories of active avoidance learning with the best fitting solution determined using the methods described above. Finally, after the best fitting models were identified, individual animal's probable class assignment was saved for further analysis in SPSS. ITRs were compared using repeated measures ANOVA with probable class assignment used as a fixed factor and total scores for ITRs for each of 5 days of training modeled as the within subjects factor.

## Results

### Study 1

Models with progressive numbers of classes were tested and compared based on the model selection criterion. Consistent improvements in fit were observed based on the information criteria (AIC, BIC, SSBIC), with diminishing reductions in scores through five classes. Class solutions demonstrated marginally significant differences in model fit through four classes based on the LMRT and significant improvement through five classes based on the BLRT compared to the four class solution. Entropy values remained in the high range across solutions. The addition of a fifth class produced an additional small class (3.3%) following an erratic pattern without significantly altering the other classes. Based on this evidence and evidence from the literature that fixed effects can lead to over-identification (Nylund et al., 2007), a four class model was retained as the most parsimonious and interpretable solution (see Table 1 in Supplementary Materials).

**Figure 1:**
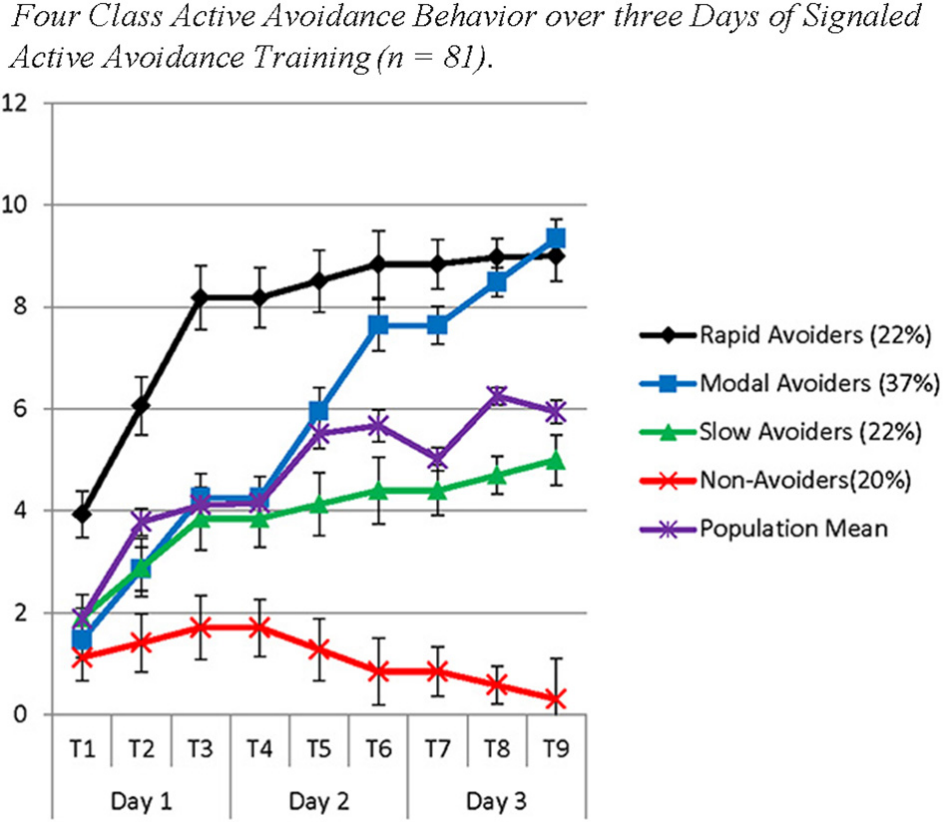
**The figure represents four latent populations identified using Latent Class Growth Analysis across three days of Signaled Active Avoidance Training**. Each time point represents the total number of successful avoidances out of 10 trials with estimates of the standard error around the mean. Each day of training is represented by three time-points. Distinct slopes for each class were identified for each day of training using a piecewise modeling approach. The purple line indicates the population mean.

**Figure 2:**
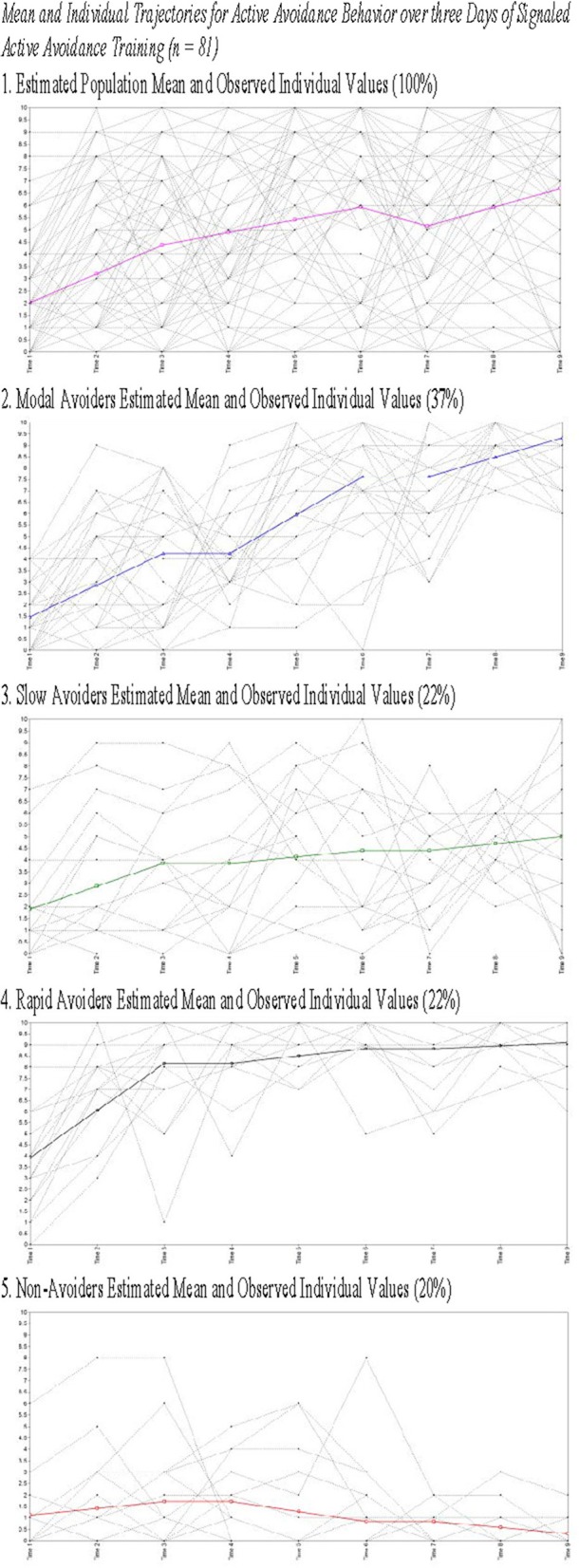
**Estimated means for and observed individual values for all animals (1) and animals grouped by class (2–5)**.

The model solution identified *four* classes with substantively distinct patterns of growth in avoidance behavior over 3 days. The best log likelihood estimates were replicated in this model indicating a global solution. Class 1 (Non-Avoiders: 20%) demonstrated an initial intercept that was significantly different from 0 (Est = 1.22; *SE* = 0.45; *p* ≤ 0.01), a non-significant slope on Day 1 (Est = 0.29; *SE* = 0.22; *p* = 0.18), a negative slope on Day 2 (Est = −0.43; *SE* = 0.18; *p* < 0.05), and a marginally significant negative slope on Day 3 (Est = −0.27; *SE* = 0.14; *p* = 0.06). Class 2 (Slow Avoiders: 21%) demonstrated an initial intercept that was significantly different from 0 (Est = 1.91; *SE* = 0.53; *p* < 0.001), and a significant increase in avoidance behavior on Day 1 (Est = 0.97; *SE* = 0.20; p < 0.001) but no increase in learning on Day 2 (Est = 0.28; *SE* = 0.37; *p* = 0.46) or Day 3 (Est = 0.29; *SE* = 0.50; *p* = 0.56). Class 3 (Modal Avoiders: 37%) demonstrated an initial intercept that is significantly different from 0 (Est = 1.47; *SE* = 0.29; *p* < 0.001) and growth in avoidance learning across all 3 days of training (Day 1: Est = 1.39; *SE* = 0.22; *p* < 0.001; Day 2: Est = 1.69; *SE* = 0.21; *p* < 0.001; Day 3: Est = 0.85; *SE* = 0.15; *p* < 0.001). Finally, Class 4 (Rapid Avoiders: 22%) demonstrated an initial intercept that was significantly different from 0 and elevated compared to the other classes (Est = 3.93; *SE* = 0.80; *p* < 0.001) along with significant positive growth on Day 1 (Est = 2.12; *SE* = 0.36; *p* < 0.001) and flat slopes for Day 2 (Est = 0.33; *SE* = 0.22; *p* = 0.13) and Day 3 (Est = 0.13; *SE* = 0.22; *p* = 0.56; see Figures 1, 2 for graphical representations of the population mean, individual trajectory means, and distribution within those trajectories). Importantly, as animals in this study were canalized, results further indicate these phenotypes could be identified even when potentially intrusive recording equipment was employed.

### Study 2

Model selection for Study 2 was identical to Study 1. The increase in sample size allowed for the estimation of free effects for the intercept and slope for day 1. All other parameters were fixed to aid in model identification. While the information criteria continued to demonstrate reductions through a five class solution, large decreases were only observed through four classes. The LMRT favored a four class solution. The BLRT continued to demonstrate improvements in fit through five classes, but once again, a five class solution revealed an additional small class (3.0%), which in this case was not substantively distinct from another identified class. Entropy values remained high across solutions. Based on these observations, a four class solution was retained (see Table 1 in Supplementary Materials).

**Figure 3:**
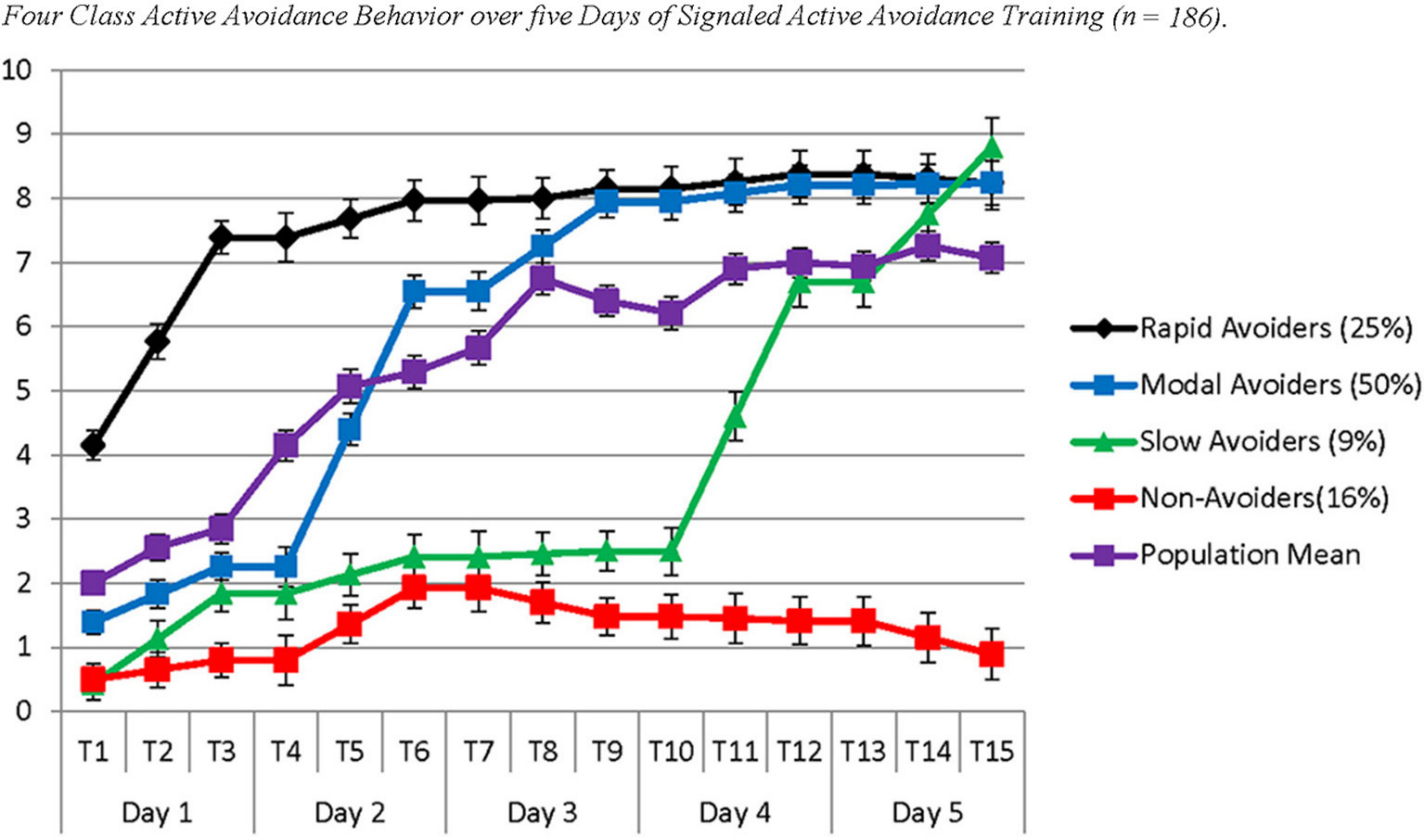
**The figure represents four latent populations identified using Latent Class Growth Analysis across five days of Signaled Active Avoidance Training and the standard error around each estimated mean for each time point within each modeled class**. Each time point represents the total number of successful avoidances out of 10 trials. Each day of training is represented by three time-points. Distinct slopes for each class were identified for each day of training using a piecewise modeling approach. The purple line indicates the population mean.

**Figure 4:**
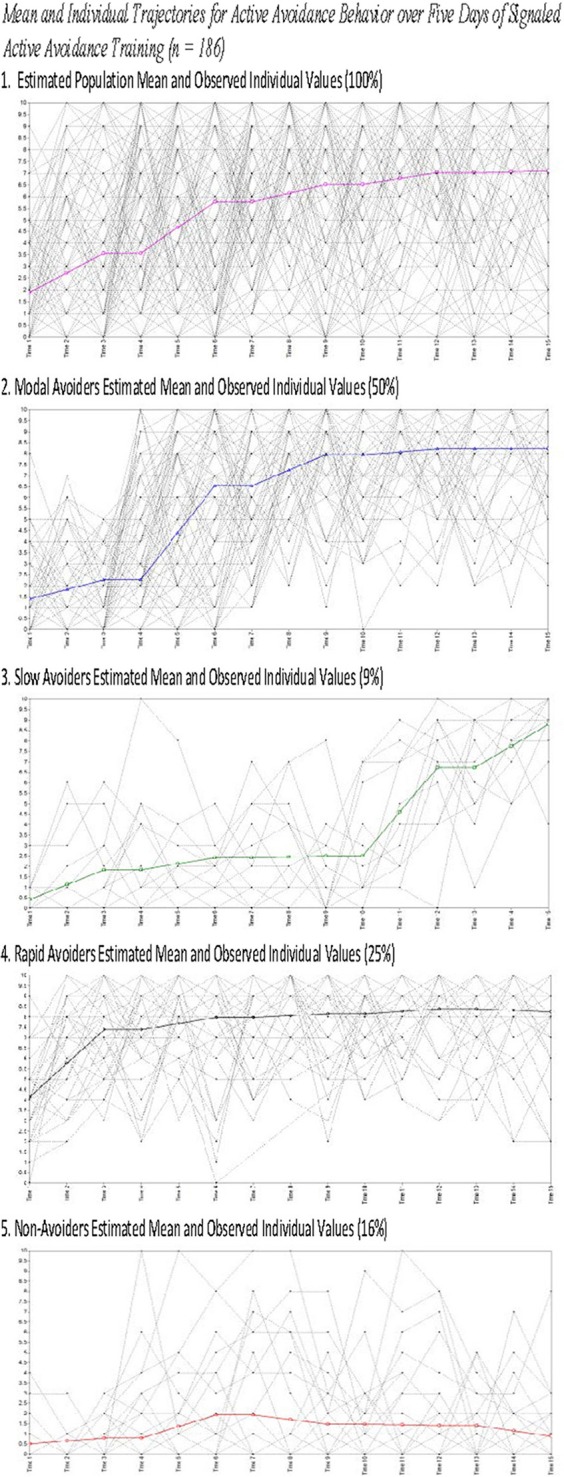
**Estimated means for and observed individual values for all animals (1) and animals grouped by class (2–5)**.

The *four* identified classes were similar to those identified in Study 1, with the noticeable exception of the Slow Avoiders, who once again demonstrated a trajectory that was distinctly higher in number of successful avoidances compared to the Non-Avoiders without large gains in avoidance learning. The best log likelihood estimates were replicated in this model indicating a global solution. In the current sample this population demonstrated rapid growth in avoidance learning through Day 4 and Day 5. Specifically, Slow Avoiders (9%) demonstrated an initial intercept that was marginally significantly different from 0 (Est = 0.43; *SE* = 0.25; *p* = 0.09), significant positive growth on Day 1 (Est = 0.70; *SE* = 0.21; *p* ≤ 0.001), non-significant growth on Day 2 (Est = 0.29; *SE* = 0.21; *p* = 0.26) and Day 3 (Est = 0.04; *SE* = 0.26; *p* = 0.87), and significant positive growth on Day 4 (Est = 2.11; *SE* = 0.14; *p* < 0.001) and Day 5 (Est = 1.04; *SE* = 0.34; *p* < 0.01). Other classes included Non-Avoiders (16%) who demonstrated an intercept that was significantly different from 0 (Est = 0.50; *SE* = 0.20; *p* = 0.01), non-significant growth on Day 1 (Est = 0.15; *SE* = 0.14; *p* = 0.29), positive growth on Day 2 (Est = 0.57; *SE* = 0.23; *p* = 0.01), and non-significant growth on Day 3 (Est = −0.23; *SE* = 0.20; *p* = 0.26), Day 4 (Est = −0.03; *SE* = 0.12; *p* = 0.79), and Day 5 (Est = −0.26; *SE* = 0.19; *p* = 0.16). Modal Avoiders (50%) demonstrated a significant intercept (Est = 1.39; *SE* = 0.16; *p* < 0.001), significant positive growth across Day 1 (Est = 0.43; *SE* = 0.12; *p* < 0.001), Day 2 (Est = 2.14 *SE* = 0.13; *p* < 0.001), and Day 3 (Est = 0.70; *SE* = 0.13; *p* < 0.001) and non-significant growth on Day 4 (Est = 0.13; *SE* = 0.09; *p* = 0.15) and Day 5 (Est = 0.01; *SE* = 0.09; *p* = 0.90). Rapid Avoiders (25%) once again demonstrated an elevated intercept compared to the other classes (Est = 4.15; *SE* = 0.39; *p* < 0.001), significant growth on Day 1 (Est = 1.62; *SE* = 0.17; *p* < 0.001), and non-significant growth on Day 2 (Est = 0.29; *SE* = 0.24; *p* = 0.23), Day 3 (Est = 0.09; *SE* = 0.11; *p* = 0.41), Day 4 (Est = 0.11; *SE* = 0.13; *p* = 0.39), and Day 5 (Est = −0.07; *SE* = 0.16; *p* = 0.68; see Figures 3, 4 for graphical representations of the population mean, individual trajectory means, and distribution within those trajectories).

#### Post-hoc analysis of inter-trial crossing

**Figure 5:**
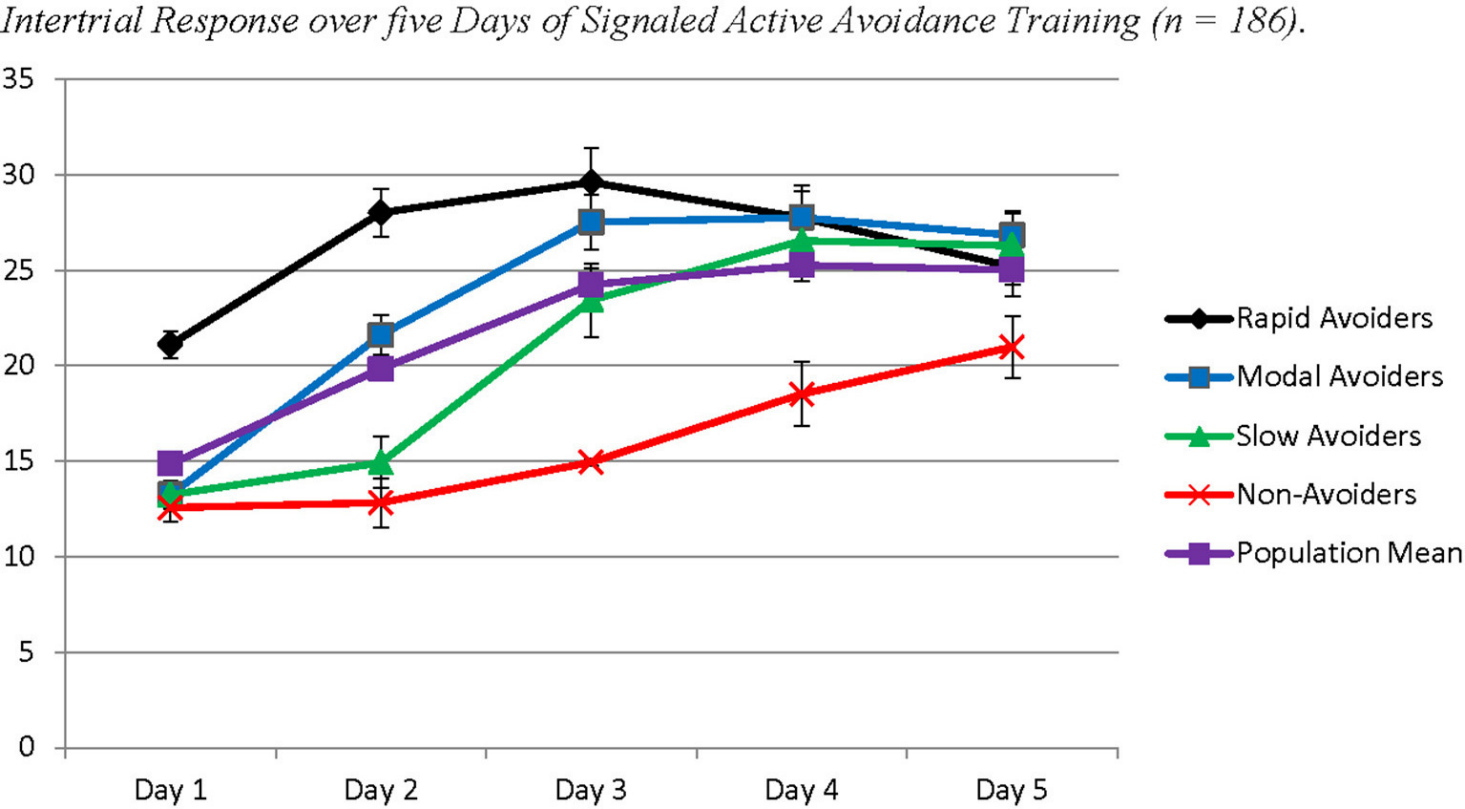
**Each time point represents the average number of inter trial crossings per day of training trial crossings per day of training**. Proportions of class membership are the same as Figure 2.

The probable class membership for individual animals from Study 2 was saved to SPSS 20 to assess differences in ITR behavior. A repeated measures ANOVA was utilized to assess the trajectory of ITRs across the 5 days, with each time point being the average ITRs for each day of training. Class membership was utilized as a between-subjects factor. This model revealed an overall effect for time (*Wilks'* λ = 0.43; *F*_(4, 172)_ = 57.94; *p* < 0.001) and a significant interaction between time and class membership (*Wilks'* λ = 0.66; *F*_(12, 455.36)_ = 6.45; *p* < 0.001). *Post-hoc* analyses using Least Squared Differences correction for multiple comparisons revealed significant differences by class on the course of ITRs that closely resembled trajectories of avoidance response (Table 2 in Supplementary Materials; Figure 5). Rapid Avoiders made significantly more ITRs early in training, whereas Non-Avoiders only began making ITRs on Day 3 of training; the other two classes were intermediate.

Finally, because the identified trajectories indicate step-like learning, means, standard deviations and confidence intervals by class for the final time point of each day of training were generated using Study 2 data. This was done to determine the distribution of the percentage of successful avoidance trials that characterizes successful AA acquisition (see Table 3 in Supplementary Materials). Results based on the mean per class and confidence intervals indicate that AA acquisition during a single day of training can be identified by successfully avoiding ≥50% of the last 10 trials and maintained through subsequent trials. Based on this observation, animals can be classified as acquiring AA in smaller samples without reliance on LGMM which comes with heavy sample size burden to identified patterns of response.

#### Post-hoc analysis of crossings during acclimation

For study 1, the number of crossings when animals were habituated to the shuttle box for 1 h prior to training was recorded. Classes were compared on baseline habituation behavior quantified as the number of crossings occurring in the 1 h prior to the initiation of training. Mean number of crossings was compared by class using a One-Way ANOVA with individual comparisons conducted using Least Squared Differences correction for multiple comparisons. Neither the overall model nor the individual comparisons approached significance. Habituation behavior was not recorded in study 2 precluding these analyses on the study 2 animals.

## Discussion

The current study identified, characterized, and replicated distinct phenotypic patterns of signaled active avoidance (AA) behavior in response to a two-way shuttling paradigm. Previous studies drawing on distinct AA populations (i.e., good vs. poor avoiders) have made important advances in understanding competition in brain circuits mediating threat and avoidance conditioning formation resulting in individual differences in instrumental behavior (Choi et al., 2010; Lázaro-Muñoz et al., 2010; Martinez et al., 2013; Moscarello and Ledoux, 2013). The current study provides empirical evidence that disaggregating distinct populations provides better model fit then a single population estimate. This indicates that disaggregating distinct populations provides more accurate estimates of animal's behavior then using the population mean which assumes a single homogenous population. Further, identifying such populations through advanced modeling methods provides substantive information about the nature of signaled AA acquisition that is of key relevance for understanding the phenomenon on all levels of investigation.

The current study presents with limitations that are relevant to the interpretation of the current results. First, while we present the weight of animals upon arrival, of key interest is their weight upon the initiation of the experimental procedures as weight may be a proxy for age and may explain heterogeneity in trajectory membership. Second, ITRs were only characterized in Study 2 data and as such the findings related to heterogeneity in ITRs were not replicated across studies. This occurred because ITRs were not consistently recorded in Study 1 limiting our ability to analyze this data. Finally, as Figures 2, 4 demonstrate, there is significant, and likely meaningful variability around the population means. The current study utilized some fixed effects to aid in model convergence though Figures 2, 4 clearly demonstrate that there is significant variability around the trajectory means. Using free effects allows for the analysis of this variability which may be relevant for addressing key questions about the identified phenotypes. For example, recent work examining trajectories of PTSD symptomatology from days after trauma exposure through 15 months found that the receipt of early exposure psychotherapy affected variability in the slope rather than predicting trajectory membership (Galatzer-Levy et al., 2013a). Such analysis can only be conducted if the slope parameter is not fixed. Despite these limitations, the current findings provide unique information about heterogeneity in behavioral responses to threat cues.

### Population characteristics of active avoidance learning

Specifically, *three* phenotypes were replicated both in terms of behavioral trajectory and relative proportion of the total population. These included Rapid Avoiders (Study 1 = 22%; Study 2 = 25%) who acquired AA within 10 trials and achieved asymptotic levels of avoidance within the *first* day of training, Modal Avoiders (Study 1 = 37%; Study 2 = 50%), who demonstrated step-like increases in AA acquisition for roughly 3 days before converging with Rapid Avoiders, and a class of animals (Non-Avoiders) that failed to acquire AA learning through both 3 and 5 days of training (Study 1 = 20%; Study 2 = 16%). In data from both studies, a Slow Avoider group was identified (Study 1 = 21%; Study 2 = 9%) that demonstrated slight gains through the *first* 3 days of training and in Study 2 demonstrated a step-like increase in avoidance acquisition across days 4 and 5.

The use of LGMM provides statistical evidence for consistent population heterogeneity in AA acquisition. Comparing the identified phenotypes to the population mean provides important insights into the limitations of assuming population homogeneity. Characterizing these populations comes with a number of benefits for future research.

The population mean provides the appearance that acquisition is linear and gradual over time, while acquisition among disaggregated phenotypes follows a step-like pattern where animals learn rapidly during some days of training and demonstrate little or no increase on other days. The use of a piecewise modeling approach with a separate slope each day of training allows for the identification of non-linear patterns of acquisition. Disaggregating populations provides evidence that the mean pattern of learning is an artifact resulting from collapsing multiple qualitatively distinct populations, including those who very rapidly acquire AA and those who consistently fail to do so. Further, related to both of the above limitations, the population mean obfuscates important information about the characteristics of signaled AA acquisition. By disaggregating populations, we can observe and characterize asymptotic levels of avoidance both prior to and following acquisition. By identifying latent populations we observe that animals en route to asymptotic levels of active avoidance successfully avoid in ≥to 50% of trials. Further, rapid acquisition appears to occur following the initiation of a new day of training indicating that memory consolidation is likely occurring between training sessions, possibly during sleep. The population mean, however, provides no clear evidence in this regard, as the mean for early trials is influenced by those who rapidly acquire and the mean of the latter trials is influenced by those who fail to acquire (see Figures 1, 2).

Analysis of inter-trial responses (ITRs) supported LGMM results in finding distinct differences between classes, with a consistent direction indicating that increased ITRs early in training facilitate AA acquisition. In particular, Rapid Avoiders were distinguished from Modal Avoiders by markedly more ITRs on Day 1 of training. Increased ITRs on Day 3 also distinguished Slow- from Non-Avoiders. This result is consistent with previous reports of decreased ITRs in an extreme group selected for poor AA and frequent freezing (Vicens-Costa et al., 2011). Given that ITRs appear to be an important predictive correlate of AA, this result also demonstrates the utility of LGMM modeling for tracking relationships between two functionally related behaviors across classes.

An alternative explanation for the identified heterogeneous trajectories of acquisition is that the Rapid, Modal, and Slow Avoider classes merely correspond to subpopulations that acquired the task on variable days of training and that number of days it takes to acquire simply reflects normal variability. Rather, what is interesting about the current results in this conceptualization is that animals have a steep curve for acquisition of active avoidance in a single day leading to asymptotic behavior, but that this acquisition can occur at different times during the training process.

### The limits of assuming and studying normality in animal behavior

The most common approach to hypothesis testing in behavioral research is *one* developed by Ronald Fischer for the analysis of shared variance between independent and dependent variables in experimental data. This approach examines the mean of the population under the assumption of normal distribution of error terms (Hald, 2007). This method and its underlying assumptions are typically adopted without significant consideration of its implications about the nature of the phenomenon under study (i.e., is it normally distributed and homogenous). These assumptions, which garner their initial evidence from the study of astronomy and botany, are based on the characteristics of the central limit theorem which state that the sum of approximately normal random variables will be a single normal distributed random variable (Stigler, 1986; Hald, 2007). However, a random variable that is the multiplicative product of multiple independent random variables, such as interactions between individual neurobiological characteristics, will not result in a Gaussian normal distribution in the dependent variable (behavior or performance) (Buzsáki and Mizuseki, 2014). This may explain evidence that learning curves for individual subjects follow abrupt step-like increases, though these effects are obfuscated by the use of a single grand population mean which provide the illusion of continuous linear patterns of learning (Gallistel et al., 2004). Step-like behavioral changes are identified using the current approach. The ability to identify these steps can facilitate the examination of time-dependent shifts in circuit functioning, which is not accessible by examining linear relationships with the grand mean or by separating a priori populations.

### Merits of utilizing statistical modeling approaches to identifying behavioral phenotypes

Studying characteristics of distinct subpopulations within animal data sets has proven highly informative, leading to identification of differences in regional neural activation (Martinez et al., 2013), and hypothalamic-pituitary axis functioning (Cohen et al., 2006). This relatively new approach has several advantages over alternative strategies for creating behavioral models, such as the use of mutant or inbred strains, which involves neurobiological differences unrelated to the behavior, rather than populations characterized solely by behavior. Utilizing population differences for modeling behavior also permits examination of multiple behaviors within each defined population—for example, threat extinction and instrumental avoidance—which, as demonstrated (Choi et al., 2010) can reveal complex latent interactions otherwise not evident from one observable behavior.

In contrast to LGMM methods used in the current and former work (Galatzer-Levy et al., 2013b), previous studies of heterogeneity in animal threat response behaviors have commonly selected subpopulations based on subjective behavioral extremes, or in some cases, used using cut-point values (Bush et al., 2007; Vicens-Costa et al., 2011; Martinez et al., 2013; Ferreira and Nobre, 2014). This approach, while still valuable, has several drawbacks. First, ignoring the relationship of extremes to the modal population obscures identification of important population distinctions that may yield useful information. For example, we were able to identify a minority population of Rapid AA avoiders as distinct from Modal Avoiders. Neglecting the modal population may also cause misinterpretation of populations as resilient or pathological; given that modal behavior is assumed to be advantageous, or at least not maladaptive (Bonanno, 2004), populations should be interpreted in this context. By identifying phenotypic populations and their parameters, such as successful acquisition being characterized ≥50% of avoidance trials or rapid avoidance being characterized by avoidance acquisition in the first day of training, LGMM results can be utilized to identify acquisition or populations in smaller datasets. This is important, as it is often impractical to conduct research on large populations of animals.

Modeling statistical heterogeneity is also valuable when analyzing the results of experimental stress interventions, such as immobilization, which are used to studying the neurobiology of stress pathology e.g., Andero et al. (2013). While examining neurobiological correlates on the aggregate following a stress induction can be informative of mechanisms underlying behavior overall, this approach also assume population homogeneity in neurobiological changes following stress induction. Given that the response to threat is generally an adaptive process (Ledoux, 2012), changes *per se* following stress are not necessarily informative of pathology. Only by identifying those who have an abnormal response can we identify mechanisms associated with stress pathology (i.e., excessive and prolonged swelling following injury, not just swelling which is normative and adaptive). More generally, any strategy that assumes homogeneity and uniformity of variance (Fox, 2008) fails to observe that heterogeneous populations may relate to the same variables in different ways, which may be highly informative—for example, thirsty people will respond differently to the presentation of a glass of water compared to those who are satiated.

The current findings and approach can significantly aid in the discovery of behavioral and neurobiological differences associated with clinically relevant learning and coping strategies. Further, the approach can be generalized to other behaviors that are of clinical importance such as appetitive responses to addictive substances. Identifying heterogeneous populations allows for the exploration of circuit functioning that differentiates populations, the identification of time dependent changes that cause shifts in behavior, and examination of manipulations that alter the proportions of the identified populations. For example, naturally occurring differences in basal CREB, a key protein required for memory formation, has been shown to be associated with distinct behavioral responses to threat conditioning training. Further, direct augmentation of amygdala CREB causes shifts from one extreme to the other (Cowansage et al., 2013).

### Neural circuitry of pavlovian and instrumental learning in AA subpopulations

Several lines of evidence suggest that impaired AA in Slow- or Non Avoiders may owe to a reduced capacity to suppress Pavlovian defensive reactions. Slow AA avoiders show increased rates of freezing (Choi et al., 2010; Vicens-Costa et al., 2011; Martinez et al., 2013), and amygdala CeA lesions both restore AA and reduce freezing. Furthermore, lesions of the infralimbic prefrontal cortex (ilPFC), which functions to inhibit CeA activation during threat extinction learning, impair AA, associated with both increased CeA activity and increased Pavlovian freezing (Moscarello and Ledoux, 2013). Therefore, as discussed by Martinez et al., naturally occurring variations in amygdala–prefrontal circuitry could underpin both poor AA acquisition and poor threat extinction. We previously reported significant heterogeneity in Pavlovian threat extinction learning, including populations of Rapid Avoiders, Slow Avoiders, and also Non-Avoiders who ultimately fail to extinguish threat (Galatzer-Levy et al., 2013b). The relationship between extinction and AA has not yet been directly determined and future studies correlating these behaviors in a common population may be highly informative.

Rapid Avoiders constituted a robustly distinct minority. It is of interest to consider neural circuits that may mediate enhanced AA acquisition. It is plausible this subpopulation has a markedly superior capacity to suppress freezing, compared to Modal Avoiders. Alternatively, or additionally, Rapid Avoiders may have enhanced motivational control of instrumental performance, or enhanced reward processing, perhaps due to more efficient neural interactions between the amygdala and nucleus accumbens (Cain and LeDoux, 2008; Boschen et al., 2011). Rapid Avoiders may also more rapidly link defensive organismic states to action, which could stem from more efficient amygdala CeA connections with cholinergic forebrain targets, previously shown to contribute to active responses to threats (Gozzi et al., 2010). It is also possible that in extremely rapid AA acquisition, where escape is reflexively elicited as soon as a route is provided, without initial freezing, escape may more closely approximate an innate survival circuit response. Finally, an equally plausible explanation is that Rapid Avoiders simply accidently crossed chambers during one of the early trials in the first session and as a result may be suppressing a freezing response without having received much exposure to the US. As such, they may have less profound Pavlovian memories to overcome and as such may not have any innate differences compared to Modal or Slow Avoiders. Thus, by identifying heterogeneous populations, hypotheses about the individual differences in experience as well as neurobiology can be examined.

### Clinical relevance of active avoidance

Identifying distinct trajectories of AA acquisition provides an inroad for translational research through comparison to quantitatively and qualitatively related patterns in clinical populations. Naturalistic studies of the course of stress pathology identify a similar heterogeneity in trajectories of symptom non-remission following trauma exposure (Bonanno et al., 2012a; Galatzer-Levy et al., 2013a). Active avoidance learning leads to robust suppression of Pavlovian defensive states, and this reduction in aversive state may further enhance instrumental learning (Choi et al., 2010; Moscarello and Ledoux, 2013). Furthermore, given that learning to terminate aversive experiences has been shown to prevent spontaneous recovery of threat response (Cain and LeDoux, 2007), learning active coping strategies may produce long term symptom management (Ledoux and Gorman, 2001). Thus, successfully acquiring AA may capture adaptive active coping responses to threat with direct relevance for understanding such responses to traumatic events (Lázaro-Muñoz et al., 2010; Martinez et al., 2013; Moscarello and Ledoux, 2013). Conversely, impaired AA may capture under-modulation of defensive states leading to the inability to instantiate behaviors that will ameliorate the threatening situation. The ability to characterize such populations in animals provides an opportunity to study neurobiological features associated with behavioral responses to manipulations such as signaled AA in a way that is not accessible in humans. The translational nature of such models can be greatly improved by identifying analogous behavioral phenotypes in both animals and humans.

### Conflict of interest statement

The authors declare that the research was conducted in the absence of any commercial or financial relationships that could be construed as a potential conflict of interest.
