# Supplementary material for: Heterogeneity in signaled active avoidance learning: substantive and methodological relevance of diversity in instrumental defensive responses to threat cues
Source: Front Syst Neurosci. 2014 Sep 24;8:179. doi: 10.3389/fnsys.2014.00179 (PMC4173321; doi:10.3389/fnsys.2014.00179)
Supplement: Supplementary file 1 [file Table1.DOCX]

Table 1

*Fit Indices for One- to-Four Class Latent Class Mixture Models of Active Avoidance Behavior*

| Fit Indices | AIC | BIC | SSBIC | | LMRT | BLRT | | Entropy | |
| --- | --- | --- | --- | --- | --- | --- | --- | --- | --- |
| Study 1(n = 81) | | | | | | | | | |
| 1 Class | 3673.79 | 3704.92 | 3663.92 | | --- | | --- | | --- |
| 2 Class | 3373.67 | 3416.77 | 3360.00 | | *p* < .01 | | *p* < .001 | | 0.96 |
| 3 Class | 3294.66 | 3349.73 | 3277.20 | | *p* = .08 | | *p* < .001 | | 0.95 |
| **4 Class** | **3248.16** | **3315.21** | **3226.90** | | ***p* = .07** | | ***p* < .001** | | **0.92** |
| 5 Class | 3208.80 | 3287.81 | 3183.74 | | *p* = .13 | | *p* < .001 | | 0.93 |
| Study 2 (n = 186) | | | | | | | | | |
| 1 Class | 12496.10 | 12573.52 | 12497.51 | --- | | --- | | --- | |
| 2 Class | 12214.30 | 12314.30 | 12216.11 | *p* < .001 | | *p* < .001 | | 0.88 | |
| 3 Class | 12089.23 | 12211.81 | 12091.45 | *p* < .05 | | *p* < .001 | | 0.93 | |
| **4 Class** | **11956.51** | **12101.67** | **11959.14** | ***p* < .01** | | ***p* < .001** | | **0.94** | |
| 5 Class | 11891.18 | 12058.92 | 11894.22 | *p* = .17 | | *p* < .001 | | 0.96 | |

*Note*: Information Criteria and model fit indices for best fitting model in **bold**. AIC= Akaike information criterion; BIC= Bayesian information criterion; SSBIC = sample size adjusted Bayesian information criterion; LMRT = Lo-Mendell-Rubin test; BLRT = bootstrap likelihood ratio test. 1- to 4-class solutions were tested with linear and quadratic parameters.
