# Supplementary material for: Heterogeneity in signaled active avoidance learning: substantive and methodological relevance of diversity in instrumental defensive responses to threat cues
Source: Front Syst Neurosci. 2014 Sep 24;8:179. doi: 10.3389/fnsys.2014.00179 (PMC4173321; doi:10.3389/fnsys.2014.00179)
Supplement: Supplementary file 2 [file Table2.DOCX]

Table 2

*Post-hoc comparisons of classes on Inter-trial crossing behavior over 5 days of active avoidance training.*

| Class (i) | Class (j) | Mean Difference (i-j) | Standard Error | *p≤* |
| --- | --- | --- | --- | --- |
| Non-Avoiders | Slow Avoiders | -4.59 | 1.58 | .01 |
|  | Modal Avoiders | -7.04 | 1.40 | .001 |
|  | Rapid Avoiders | -10.38 | 1.53 | .001 |
| Slow Avoiders | Modal Avoiders | -2.46 | 1.44 | .09 |
|  | Rapid Avoiders | -5.79 | 1.57 | .001 |
| Modal Avoiders | Rapid Avoiders | 3.34 | 1.39 | .05 |
| *Note*: Post-hoc comparisons utilized least squares differences to correct for multiple comparisons. | | | | |
