# Supplementary material for: Heterogeneity in signaled active avoidance learning: substantive and methodological relevance of diversity in instrumental defensive responses to threat cues
Source: Front Syst Neurosci. 2014 Sep 24;8:179. doi: 10.3389/fnsys.2014.00179 (PMC4173321; doi:10.3389/fnsys.2014.00179)
Supplement: Supplementary file 3 [file Table3.DOCX]

Table 3

*Means, Standard Deviations, and Confidence Intervals by Class of the Percentage of Successful Avoidance Trials for the Last 10 Trials Per Day of Training.*

|  | Mean (SD) | Confidence Interval |
| --- | --- | --- |
| Avoidance Percentile T3 |  |  |
| Rapid Avoiders | 73.04 (20.64) | 66.91-79.17 |
| Modal Avoiders | 15.53 (16.44) | 12.17-18.90 |
| Slow Avoiders | 12.86 (20.16) | 1.21-24.50 |
| Non-Avoiders | 7.10 (9.38) | 3.66-10.54 |
| Avoidance Percentile T6 |  |  |
| Rapid Avoiders | 79.15 (17.30) | 67.91-83.58 |
| Modal Avoiders | 57.87 (31.86) | 51.35-64.40 |
| Slow Avoiders | 17.86 (13.11) | 10.29-25.43 |
| Non-Avoiders | 16.00 (24.01) | 7.03-24.97 |
| Avoidance Percentile T9 |  |  |
| Rapid Avoiders | 80.00 (20.69) | 73.71-86.29 |
| Modal Avoiders | 79.89 (20.24) | 75.75-84.04 |
| Slow Avoiders | 17.86 (22.25) | 5.01-30.70 |
| Non-Avoiders | 13.87 (20.44) | 6.37-21.37 |
| Avoidance Percentile T12 |  |  |
| Rapid Avoiders | 83.64 (19.54) | 77.70-89.58 |
| Modal Avoiders | 81.38 (17.57) | 77.78-84.98 |
| Slow Avoiders | 66.43 (27.63) | 50.48-82.38 |
| Non-Avoiders | 17.42 (24.22) | 8.54-26.30 |
| Avoidance Percentile T15 |  |  |
| Rapid Avoiders | 82.17 (21.59) | 75.76-88.59 |
| Modal Avoiders | 81.06 (17.13) | 77.56-84.57 |
| Slow Avoiders | 88.57 (16.57 | 79.00-98.14 |
| Non-Avoiders | 10.34 (17.21) | 3.79-16.89 |
| *Note*: SD = Standard Deviation; Shading indicates trials after acquisition of avoidance behavior by class. | | |
